# Early Interventions to Prevent Post-Traumatic Stress Disorder in Youth after Exposure to a Potentially Traumatic Event: A Scoping Review

**DOI:** 10.3390/healthcare10050818

**Published:** 2022-04-28

**Authors:** Hala Kerbage, Ola Bazzi, Wissam El Hage, Emmanuelle Corruble, Diane Purper-Ouakil

**Affiliations:** 1 Department of Child and Adolescent Psychiatry, Saint Eloi University Hospital, 34090 Montpellier, France; 2 Center for Epidemiology and Population Health (CESP), INSERM U1018, Developmental Psychiatry Team, Paris-Saclay University, Villejuif CEDEX, 94807 Paris, France; 3 Faculty of Health Sciences, American University of Beirut, Beirut 1107 2020, Lebanon; oab09@mail.aub.edu; 4 Center of Clinical Investigation iBrain 1253, University of Tours, 37032 Tours, France; wissam.elhage@univ-tours.fr; 5 CESP, MOODS Team, INSERM U1018, School of Medicine, Paris-Saclay University, Kremlin Bicetre, 94275 Paris, France; emmanuelle.corruble@aphp.fr; 6 Department of Psychiatry, Paris-Saclay University Hospital, Kremlin Bicetre, 94275 Paris, France; d-purper_ouakil@chu-montpellier.fr

**Keywords:** trauma, PTSD, children and adolescent, early intervention, prevention, potentially traumatic events (PTEs), coping strategies, psychoeducation, traumatic exposure, parental support

## Abstract

The worldwide occurrence of potentially traumatic events (PTEs) in the life of children is highly frequent. We aimed to identify studies on early mental health interventions implemented within three months of the child/adolescent’s exposure to a PTE, with the aim of reducing acute post-traumatic symptoms, decreasing long term PTSD, and improving the child’s adjustment after a PTE exposure. The search was performed in PubMed and EMBASE databases resulting in twenty-seven articles meeting our inclusion criteria. Most non-pharmacological interventions evaluated had in common two complementary components: psychoeducation content for both children and parents normalizing early post-traumatic responses while identifying post-traumatic symptoms; and coping strategies to deal with post-traumatic symptoms. Most of these interventions studied yielded positive results on outcomes with a decrease in post-traumatic, anxiety, and depressive symptoms. However, negative results were noted when traumatic events were still ongoing (war, political violence) as well as when there was no or little parental involvement. This study informs areas for future PTSD prevention research and raises awareness of the importance of psychoeducation and coping skills building in both youth and their parents in the aftermath of a traumatic event, to strengthen family support and prevent the occurrence of enduring post-traumatic symptoms.

## 1. Introduction

The worldwide occurrence of potentially traumatic events (PTEs) in the life of children and adolescents is highly frequent, including maltreatment, neglect, abuse, bullying, as well as war, displacement, and armed conflict [1,2,3,4,5]. In a survey involving 24 countries across the globe, the World Mental Health (WMH) reported that 70% of the populations were exposed at least once in their life to a PTE, with higher rates of exposure among children, adolescents, and young adults [6]. Moreover, Post-traumatic stress disorder (PTSD) is shown to develop more frequently when the traumatic events occur during childhood and adolescence [7,8,9].

Most studies estimate that spontaneous recovery for both adults and youth after exposure to PTEs is the norm and that most trauma-exposed individuals manifest various levels of acute post-traumatic stress reactions and then regain their baseline functioning over several weeks [10,11]. However, complex individual and contextual factors as well as the type of the PTE itself might lead to a failure of recovery, since a substantial 6–20% of individuals go on to develop PTSD [12,13,14]. Given the number of youths who encounter PTEs each year worldwide, the risk of developing PTSD is still high [15,16], leading to interpersonal and educational challenges as well as a significant impact on the child and family functioning [17,18,19]. Moreover, exposure to adverse and traumatic childhood experiences increases the risk of both physical and mental illnesses as well as substance use disorders in adult life, leading to reduced social and economic opportunity and impaired role functioning [20,21,22,23,24].

Emerging evidence on the dysfunctional brain circuits underlying PTSD focuses on the role of altered brain activity and connectivity in the fear extinction process that is commonly found in PTSD [25,26,27,28,29,30]. More specifically, a smaller volume and altered activity patterns in the ventromedial region of the prefrontal cortex (vmPFC) have been observed in patients with PTSD, suggesting the implication of frontal lobe circuitry in altered fear extinction features [25,26]. In a recent study conducted on patients with lesions in the ventromedial portion of the prefrontal cortex, the authors reveal that the vmPFC is involved in the acquisition of emotional conditioning, and assess how bilateral lesions of the vmPFC compromise the generation of a conditioned psychophysiological response during the acquisition of threat conditioning (i.e., emotional learning) which may lead to the perpetuation of PTSD and anxiety symptoms [25]. Another recent theoretical review discussed the distinct yet fundamental role of anterior/posterior subregions of the vmPFC in the processing of safety-threat information and in the evaluation and representation of stimulus-outcome’s value needed to produce sustained physiological responses [26]. These functional and structural alterations of the neural networks that seem to underlie fear conditioning and learning, particularly in PFC, might contribute to the complex etiology of anxiety and traumatic stress syndromes and have important treatment implications [31].

The high rates of traumatic exposure and associated clinical and neurobiological manifestations, along with an elevated public health burden, indicate the importance of early efficient interventions at a secondary prevention level, that address acute posttraumatic stress reactions in the aftermath of a traumatic event and aim at decreasing the risk of developing long-term PTSD while increasing recovery and adjustment after exposure to a traumatic event [10,32,33,34].

Current international guidelines for early and acute post-traumatic interventions shift recommendations away from psychological debriefing and toward Psychological First Aid (PFA) [35,36]. However, evidence is lacking regarding the efficiency of PFA in preventing long term PTSD, despite being often recommended as a first line intervention in various trauma guidelines, especially in humanitarian settings and complex emergencies [37,38]. Psychological First Aid was developed by the World Health Organization with the aim of providing affordable first-line psychosocial support by any lay person participating in relief efforts for populations affected by complex emergencies within hours or days of the onset of traumatic events [39]. It is, therefore, a psychosocial intervention rather than a clinical one, and its aim is to provide safety and protection from further harm, assist in accessing basic needs, offer non-intrusive support, and link to available services and social support systems [37,39]. Although it seems relevant to implement it as immediate psychosocial assistance in acute settings, instead of psychological debriefing, it does not seem to be enough on its own as an early intervention to prevent the later occurrence of PTSD.

Controlled clinical trials among adults recently exposed to a PTE find that the risk of PTSD can be decreased by cognitive behavioral treatments targeting symptom reduction and the enhancement of adaptive coping strategies [40,41]. In youth, however, data on secondary prevention of trauma-related disorders are scarce. Epidemiological studies on risk and protective factors for PTSD in children and adolescents show that in the early periods following exposure to a traumatic event, family and social support are crucial in contributing to children’s adjustment and coping with posttraumatic stress and in decreasing the risk of developing PTSD [42,43,44]. Family support consists of being actively present and available to the child following the onset of the traumatic event, modeling to the child efficient coping, successfully maintaining routines and stability, and offering protection and reassurance as needed [45].

Other protective factors established are the quality of family relationships, particularly caregiver-child relationships, as well as child and caregiver emotional regulation skills [45,46]. Parents who can support effective coping through modeling and coaching, while being attentive to their children’s needs and concerns, facilitate children’s adjustment following a PTE exposure [45,47,48]. All these protective factors could be the targets of an early intervention to prevent PTSD in youth and highlight the need for clinical treatments complementary to PFA, that increase parental coping strategies and shift the focus from pure symptom reduction to skill development in secondary prevention objectives during the peri-traumatic period. In this review, we aimed to identify studies on early mental health interventions (pharmacological and non-pharmacological) implemented within three months of the child/adolescent’s exposure to a PTE, with the goal of decreasing acute post-traumatic symptoms and the risk of long-term PTSD.

## 2. Methods

### 2.1. Eligibility Criteria

We searched for articles that met the below inclusion criteria:

*Population of interest.* Children and adolescents (ages 1 to 20 years old) were assigned to mental health interventions aiming at the secondary prevention of PTSD and provided within 3 months of the child or adolescent’s exposure to a traumatic event. Interventions targeting adults aged 20 and above were excluded from the study. Interventions occurring more than 3 months after the exposure to a PTE were excluded from the study. Articles reporting on interventions targeting ongoing trauma (war/conflict/violence) were included in the review.

*Event of interest*. Potentially traumatic events included burn injuries, falls and sporting injuries, motor vehicle accidents, sexual or physical abuse, medical events, procedures, or treatments, natural disasters, war/conflict/violence, and various accidental and unintentional injuries.

*Types of intervention.* There were no restrictions on the type of mental health interventions provided, which ranged from pharmacological interventions to psychosocial and structured cognitive-behavioral interventions, among others.

*Study design*. There were no restrictions made on the design of the study.

We only included articles that were published in the English or French languages.

### 2.2. Information Sources

The electronic databases searched were PubMed and Embase. No restrictions were made on the dates of article publication. The research strategy was developed with a professional university librarian who assisted us in the exportation of retrieved records. We used both Medical Subject Headings (MeSH) terms and free-text words. We searched PubMed by using Medical Subject Headings (MeSH) terms and included the terms Stress Disorders, Post-Traumatic, prevention and control with the filters: “Child: birth-18 years”, “Adolescent: 11–20 years”. We searched Embase by using keywords and included ‘post-traumatic stress disorder”, with filters excluding Medline, and restricted to adolescents, children, infants, and newborns. No other restrictions were applied while conducting the search. The search yielded 1268 articles, with 27 articles meeting the inclusion criteria.

### 2.3. Study Selection

The research team developed screening guides for the specific purpose of this review, that were used by two team members to screen independently and in duplication, both the titles and abstracts of the identified citations, as well as the full text. Full texts were retrieved based on the title and abstract screening whenever it was evaluated as eligible by at least one of the two reviewers. A third reviewer from the team member was consulted anytime there was a disagreement regarding the full-text screening by the two main reviewers.

### 2.4. Data Abstraction

The research team initially developed standardized data abstraction forms that were tested and then used by the reviewers to abstract data from selected articles. Research characteristics abstracted included type of intervention, study design, intervention components, the timeframe between the exposure to the PTE and the intervention, type of traumatic event, target population characteristics, age of target population, targeted outcomes, and findings. We specifically looked at the rates of post traumatic symptoms and measures of coping/adaptive strategies following the interventions tested.

## 3. Results

**Figure 1:**
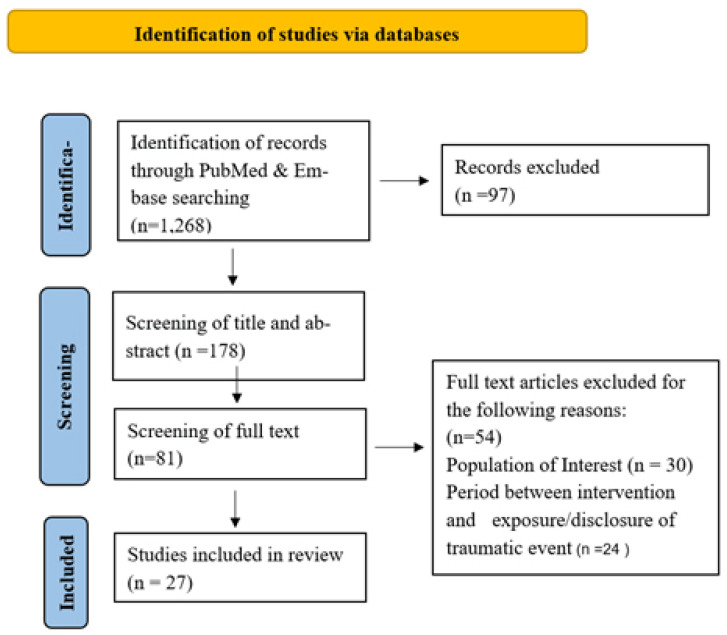
PRISMA Flowchart.

The findings of our search are presented according to the ‘Preferred Reporting Items for Systematic Review and Meta-Analysis Protocols’ (PRISMA) flowchart (Figure 1). The search on PubMed and Embase yielded 1268 records, and we retained 178 for the title and abstract screening. Out of those, 97 records did not meet our eligibility criteria, leaving 81 records for full-text screening, out of which 54 records were excluded for not satisfying the eligibility criteria (different population of interest, interventions occurring after more than 3 months of the trauma incident). Thus, only 27 papers were retained for analysis and review.

### 3.1. Research Characteristics

The research characteristics and main findings for each article are presented in Table 1 and Table 2. The design of the studies included 20 randomized control trials, three experimental studies, one prospective longitudinal study, one meta-analysis, one case report, and one retrospective review of medical records. Interventions implemented in the studies included pharmacotherapy, Child and Family Traumatic Stress Intervention (CFTSI), music therapy, structured cognitive-behavioral intervention, information provisioning, stepped preventive care, web-based early intervention and psycho-educational intervention, provision of coping skills and coaching strategies, psychoeducation, structured debriefing process, psychosocial interventions consisting of cognitive-behavioral techniques and various other techniques, hypnosis, in addition to massage and humor therapy. The time of intervention after the traumatic event ranged between 6 h and 60 days. Four studies had interventions carried out during an ongoing traumatic event, which was either war or violent conflict. Traumatic events included burns, falls, motor vehicle accidents, sexual and physical abuse, sports injuries, physical assaults, animal bites, surgery, acute medical events, natural disasters, violence, and war. The target population’s age ranged from 12 months to 20 years old.

### 3.2. Pharmacological Interventions

Seven studies explored the efficiency of early pharmacotherapy in reducing the psychological impact of traumatic events [49,50,51,52,53,54,55]. The traumatic events that required intervention were burn injuries [51,52,53,54,55], and physical [50] or unintentional injuries [49]. Intervention components included opioid administration [49], propranolol treatment [50,51,52], morphine administration [52,55], and sertraline administration [54]. Outcomes targeted were PTSD [49,50,51,52,53,54,55], in addition to anxiety and depression [51]. Three studies were double blind randomized control trials [50,51,54] in which the control group received a placebo in place of the treatment under study [50,54] or a different type of intervention (non-propranolol treatment) [51]. Opioid medication overall, and morphine specifically, was evaluated in three studies based on previous research showing that increased endorsed pain is positively associated with current and future PTSS [49]. Opioid administration did not prove to mediate the association between the pain endured and post-traumatic stress (PTS) [49], while one study proved the effectiveness of morphine in decreasing PTSS, especially arousal symptoms [55]. The other study proved a positive association between the dose of morphine and reduction in PTSD symptoms [52]. Sertraline was another drug shown to be moderately effective in preventing PTSD symptoms in comparison to placebo. However, the change was only significant in symptoms reported by parents and not in symptoms perceived by the children themselves [54].

### 3.3. Non-Pharmacological Interventions

#### 3.3.1. Child and Family Traumatic Stress Intervention (CFTSI)

Two studies examined the efficiency of the CFTSI in reducing the psychological impact of traumatic events [10,56]. The traumatic events that required the intervention were different in nature and included among others, physical assaults [10,56], injuries [10], witnessing violence [10], animal bites [10], motor vehicle accidents [10], sexual abuse [10,56], and threats of violence [10]. Intervention components included sessions differently administered to the caregiver alone, the child alone, and the caregiver and child together. CFTSI sessions included various components, such as psychoeducation, case management, providing support and strengthening coping skills, improving child-caregiver communication, and normalizing symptoms and feelings [10]. Outcomes targeted were PTSD [10,56], and anxiety [10]. Both studies (one RCT and one multi-site meta-analysis) revealed that CFTSI had a positive impact on reducing PTSD following exposure to a PTE. The RCT showed that the intervention group presented fewer PTSD diagnoses in comparison with the control group and had significantly lower anxiety scores 3 months following the intervention [10]. In the other study, the authors used a multi-site meta-analytic approach to evaluate pooled and site-specific therapeutic effect sizes of the CFTSI for both caregivers and children, based on data from 10 community treatment sites trained in CFTSI. Findings reveal that CFTSI was significantly correlated with reductions in PTS in children, but also with significant improvements in post-traumatic stress scores in 62% of caregivers participating in the study [56].

#### 3.3.2. Music Therapy

One RCT investigated the effect of music therapy on reducing the psychological impact of traumatic events compared to a control group receiving standard care [57]. The traumatic event that required the intervention was hematopoietic stem cell transplants (HSCT). The intervention follows a patient-centered approach where children choose the musical instrument and are solicited to interact by singing and listening with the therapist. The sessions may remain wordless, or the child may choose to express any emotions or sensations that emerge. The targeted outcome was physiological parameters (heart rate, blood pressure, oxygen saturation) used as a measure of stress level and post-traumatic arousal symptoms. The study revealed that the intervention lowered the heart rate of participating children, suggesting a decreased stress level, and potentially lower risk of PTSD development according to the authors 4 to 8 h after the intervention [57].

#### 3.3.3. Structured Cognitive-Behavioral Intervention

One experimental uncontrolled study explored the effect of cognitive-behavioral intervention in reducing psychological harm following exposure to a tsunami in Thailand [58]. Intervention components included teaching children how to manage post-traumatic stress symptoms using cognitive behavioral techniques. The targeted outcome was the measure of post-traumatic symptoms using the Children’s Impact of Events Scale (CRIES-13). Findings revealed that the CRIES score significantly decreased when the children were already prone to PTSD [58], and significantly increased in other children 2 weeks after the intervention.

#### 3.3.4. Psychosocial Interventions

Three cluster RCTs [59,61,62] and one RCT [60] examined the effect of school-based, group format psychosocial interventions [59,61,62] and a family focused psychosocial intervention [60] on reducing psychological harm from war/conflict [59,61,62] and political violence [62]. Intervention components included cognitive-behavioral techniques [59,61], creative-expressive, experiential therapy, and cooperative play [59,61], leadership skills program, cinema clips, relaxation technique scripts [60], trauma-processing activities, and creative-expressive elements [62]. Outcomes targeted included PTSD [59,60,61,62], depressive symptoms [59,60,61,62], anxiety symptoms [59,60,61,62], psychological difficulties [59,61,62], function impairment [59,62], conduct problems [60], trauma idiom [62], aggression [62], coping [62], social support [62], and family connectedness [62]. A psychosocial intervention based in the schools in the context of Nepal’s conflict indicated moderate beneficial effects in the short-term among the intervention group characterized by reduced aggression among boys and increased prosocial behaviors among girls, along with an increased sense of hope reported by older children. However, there was no reduction in posttraumatic symptoms [59]. An intervention with youth at risk of attack and exposed to war in the north-eastern Democratic Republic of Congo, and focusing on families, demonstrated decreased post-traumatic symptoms among participants in comparison to controls, in addition to significant improvements in depressive and anxiety symptoms, a moderate increase in pro-social scores, and moderate-large decrease in conduct problems 3 months after the intervention [60]. A study examining the effects of a preventive mental health school-based intervention for children living in war-affected Sri Lanka did not report any effects on primary outcomes of intervention after 1 week and 3 months besides conduct problems which were stronger for younger children and specific subgroups. Additionally, when children experienced fewer ongoing war-related stressors, the effects of the intervention were stronger on anxiety, PTSD, and functional impairment [61].

In a study among school aged children living in communities affected by violence in Indonesia, the intervention evaluated led to reductions in posttraumatic stress symptoms and helped instill self-reported hope after 1 week and 6 months. However, it did not lead to a decrease in traumatic-stress associated symptoms, functional impairment, or internalizing symptoms [62].

#### 3.3.5. Hypnosis

One study of four case reports explored the effect of hypnotic therapy on reducing psychological impacts following exposure to a traumatic physical injury [63]. Intervention components included different hypnotic techniques. The targeted outcome was PTSD. The study revealed that patients clinically improved after several sessions of hypnosis [63]. It was not clearly presented how PTSD was measured or how symptoms were assessed as improvements were either self-reported by the patients or reported by the physicians. Throughout the cases, it is mentioned how patients reported better sleep without assisting medications, fewer nightmares and flashbacks.

#### 3.3.6. Psychological Intervention

One RCT examined the efficiency of a single-session psychological intervention in decreasing psychological harm in children aged 7–16 recently exposed to road traffic accidents at 2 or 6 months post-intervention [64]. The intervention was a four step process that included reconstructing the accident, creating a narrative of the trauma, identifying appraisals related to the accident, and psychoeducation. A psychologist then presented helpful strategies and advice on dealing with acute stress reactions. The outcomes targeted included PTSD symptoms, depression, and behavior. The findings revealed no significant differences in post-traumatic symptoms, however, the intervention demonstrated effectiveness in decreasing depressive symptoms as well as externalized behaviors in preadolescent children [64].

#### 3.3.7. Complementary Intervention

One RCT investigated the efficiency of a complementary intervention in decreasing psychological harm following stem cell transplantation (SCT) as opposed to standard care at week + 24 [65]. A total number of 171 children and their parents were recruited from four different sites and assigned randomly to either a child-focused intervention; a family intervention; or standard clinical care. The child focused intervention consisted of massage and humor therapy; the family intervention included relaxation/imagery. Depression, posttraumatic stress, HRQL, and benefit finding, were the targeted outcomes measured. The study revealed significant improvements in all outcomes for all three groups and there were no significant differences between the three intervention groups [65].

#### 3.3.8. Debriefing

One RCT examined the effect of debriefing on reducing psychological harm following exposure to road traffic accidents [66]. The intervention was manualized and researchers guided the child through a structured debriefing where the trauma was reconstructed. The child was encouraged to express thoughts and emotional reactions and was given information on how to cope. The targeted outcomes were self-reported measures of psychological distress and diagnostic criteria for PTSD The study revealed no significant gains in the debriefing group in comparison to the control group at 8 months follow-up [66].

#### 3.3.9. Information Provisioning Intervention

Two RCTs examined the effect of information provisioning on reducing psychological harm following motor vehicle accidents [67], falls [67], sporting injuries [67], and accidental injuries [68]. Intervention components included booklets provided to parents [67,68], and children [68], aiming at normalizing traumatic stress response [67,68], and reliving trauma reactions through different strategies [68]. Outcomes targeted were anxiety and PTS symptoms in children and parents [67], and long-term PTS reactions [68]. One of the studies assessing outcomes after 1 and 6 months revealed a decrease in overall PTSS, parental posttraumatic intrusion symptoms, and anxiety symptoms among children in the intervention group [67]. The other study revealed that children in the control group had significantly higher post-traumatic symptoms at 6-month follow-up compared to children in the intervention group only when initial emotional distress was elevated [68].

#### 3.3.10. Psychoeducation and Coping Interventions

Three RCTs examined the effect of psychoeducation [69,70,71], while three RCTs [72,73,74]–of which one multi-site RCT [72] and one cluster RCT [74] examined the effect of coping interventions, on reducing psychological harm following unintentional injuries [69,70,72,74], medical events [73,74], pediatric injuries [71], and other causes [74]. Psychoeducation components used included a booklet for parents and a web page for children aimed at normalizing and relieving trauma responses [69]; two sessions that incorporated psychoeducation and semi-structured interviews [70]; and practical information integrated into psychoeducation and methods for parents to assist children during post-injury [71]. Outcomes targeted were PTSD [69,70,71], anxiety [69,70], and depression [40,41]. One of the studies revealed that children in the intervention group manifested an improvement at 4–6 weeks and 6 months follow-up in anxiety symptoms, as well as a reduction in trauma symptoms in children with higher baseline trauma scores, while children in the control group had worsening symptoms [69]. However, the other two studies revealed that there was no reduction in depression [70], anxiety, PTSD [70,71], or an increase in health-related quality of life [70], or parent knowledge [42] in the intervention arm as opposed to control groups at follow-up at 6 months [70] and 6 weeks follow-up [71].

The other studies incorporated different components of coping, such as narrative [72], developmentally appropriate resources, and games [72,73], psychoeducation [72,74], and coping strategies [72]. Targeted outcomes were PTSD symptoms [72,73], and severity [72], PTSD diagnosis [72], functional impairment [72], behavioral difficulties at different points in time [72], maternal anxiety and beliefs [74], depression [74], parental stress [74], parent involvement in children’s care [74], child adjustment [74]. One of the studies revealed a significant impact of the intervention on post-traumatic stress severity over time, PTSD diagnosis, functional impairment, and behavioral problems at 3- and 6-months follow-up [72]. The second study, which was online self-directed in nature also revealed that the intervention could have a preventive persistent effect on posttraumatic stress after 6 or 12 weeks [73]. The third study assessing outcomes at 1, 3, 6, and 12 months and which was intended for mothers and children revealed positive functional and emotional coping outcomes among mothers resulting in decreased adjustment problems in children [74].

## 4. Discussion

All articles selected for review were published after the year 2000, confirming that the subject of early intervention in the peritraumatic phase to prevent the occurrence of PTSD among children and adolescents is a relatively new and understudied phenomenon, in contrast with the abundant publications on the treatment of chronic PTSD. Our review included mental health interventions implemented within three months of a PTE, with the aim of preventing PTSD and improving the child’s adjustment and functioning after a PTE exposure. We found 27 articles that met our eligibility criteria, of which seven studies evaluated pharmacological interventions and twenty studies assessed non-pharmacological interventions.

### 4.1. Pharmacological Interventions

Regarding pharmacological interventions, Sertraline was shown to be moderately effective in preventing PTSD symptoms in comparison to placebo on parent-reported symptoms [54], while morphine administration in the acute phase was shown to be associated with decreased PTSS arousal symptoms [55]. However, the results regarding morphine should be carefully interpreted, since it was administered in the context of burn injuries, and should not be generalized to all types of PTE. More specifically, morphine might play a preventive role by reducing endorsed pain caused by the burn injury, which was shown to be associated with a decrease in current and future PTSS [49]. Propranolol, however, was not shown to be efficient in reducing PSS or preventing PTSD in two RCTs and one retrospective review of medical records [50,51,53]. A deeper understanding of fear learning neural networks involved in traumatic exposure and PTSD may contribute to the advancement and implementation of alternative treatments for traumatic stress symptoms [31]. For example, a recent review described the potential and effectiveness of non-invasive brain stimulation (NIBS) to interfere and modulate the abnormal activity of neural circuits (i.e., amygdala-mPFC-hippocampus) involved in the acquisition and consolidation of fear memories, which are altered in PTSD, depression and anxiety disorders [75]. Similarly, another recent study illustrated the therapeutic potential of NIBS as a valid alternative in the treatment of abnormally persistent fear memories that characterize patients with anxiety disorders that are resistant to psychotherapy and/or drug treatments [76]. Although this therapeutic technique might be more relevant in the context of enduring PTSD symptoms rather than as an early intervention in the peritraumatic phase, it is important to gain a better understanding of the alterations in neurobiological as well as endocrinological activity that can be a target for more precise and individualized innovative treatments [77]. In this regard, alterations in the hypothalamic-pituitary (HPA) axis and the involvement of inflammation seem to be implicated in the pathophysiology of PTSD [77,78] and might be a target for biological treatment. In a recent meta-analysis of hydrocortisone as a potential preventive or curative treatment for PTSD, hydrocortisone appears to be a promising and efficient low-cost medication for the prevention of PTSD among adults but there are no available studies among children and adolescents

### 4.2. Non Pharmacological Interventions

In line with research on protective factors supporting recovery following a traumatic event, which asserts the role of family support [45,46,47,48], most non-pharmacological interventions evaluated involved parents as well as children [10,56,60,64,65,67,68,69,70,71,72,73,74]. Those interventions had in common the emphasis on two distinct but complementary components: psychoeducation content for both children and parents on trauma reactions, normalizing early post-traumatic responses while identifying post-traumatic symptoms and coping strategies to deal with post-traumatic symptoms through cognitive behavioral techniques and relaxation techniques. Most of these interventions studied yielded positive results on outcomes with a decrease in post-traumatic symptoms as well as anxiety and depression. However, Kassam-Adams et al., in an RCT examining the prevention of PTSD based on psychoeducation around incorporated into pediatric care [70], found no reduction in PTSD or depression severity, which reveals that psychoeducation alone may not be sufficient to prevent PTSD, especially since it was two-session interventions only. The other RCTs that had negative results evaluated psychosocial interventions in the context of ongoing traumatic events (war and armed conflicts) [59,61,62] and did not include parents as active participants in the intervention. These negative results may be due to the context of ongoing traumatic stressors and war, where international recommendations on mental health in complex crisis settings emphasize community-based approaches that help face everyday stressors rather than clinical, individual-based approaches focused on post-traumatic symptoms [36,79,80]. With this particular type of PTE (war and armed conflicts), mental health interventions that seem to be needed to buffer the effects of traumatic events are interventions that strengthen community and family support and networks, social engagement and help regain a sense of purpose amidst ongoing adversity.

One intervention that looks promising is the CFTSI, a brief early intervention elaborated at the Yale Child Study Center for children seven years old and older who have recently experienced a potentially traumatic event (PTE). CFTSI is a five to eight-session family-focused model that aims to strengthen parents’ support of the child by facilitating the identification of common child reactions to potentially traumatic events, improving communication between the child and caregiver, and teaching the caregiver and child coping strategies and behavioral interventions to decrease acute posttraumatic reactions.

The model has been implemented with children who have been exposed to various PTEs, including sexual abuse, domestic and community violence, motor accidents, animal bites, and other injuries [10,56] but not in the context of war and political violence. CFTSI can be implemented shortly after the exposure to a PTE or in the context of later disclosure of sexual abuse, which can trigger the emergence of post-traumatic reactions. Berkowitz et al., in an RCT comparing CFTSI with a five-session psychoeducational and supportive counseling model, found that children in the CFTSI group presented fewer full and partial PTSD diagnoses in comparison with the control group 3 months following the intervention [10].

Even more importantly, a multi-site meta-analysis studying the efficiency of CFTSI in various centers indicated significant improvement of post-traumatic symptoms in adult caregivers who participated in CFTSI with their children [56]. This is one of the few studies that specifically evaluated the effect of early intervention after a child’s exposure to a PTE on parental symptoms and parental mental health, along with the COPE Intervention study [74], a mental health intervention provided to youth who are critically ill and their mothers, based on psychoeducation and coping. In this study, mothers in the intervention group reported increased maternal functional and emotional coping resulting in better adjustment in the child [74]. These findings are in line with research establishing correlations between the psychopathology of parents and that of children since family environment and parental functioning systematically influence the association between exposure and outcome for children. Moreover, the implication of events that affect their children, can traumatically affect parents themselves which may influence their capacity to efficiently provide their parental role to a child rendered vulnerable after a PTE exposure [56,81]. Parents going through high distress are less capable of displaying a sense of stability and safety to the child, and of supporting the child in progressing [82,83]. When parents are coping well themselves, however, this can facilitate the child’s adjustment after a PTE exposure, through modeling efficient coping skills, maintaining balance through routine and regulation, and instilling self-efficacy and relatedness [45,84]. This emphasizes the importance of interventions to reinforce parental capacities in the aftermath of the child’s exposure to a traumatic event and offer additional support to parents who are highly affected by the exposure of their child to a traumatic event, especially since parents’ abilities to support their children are related to their own distress level [85]. Other members of the family, such as siblings, seem to influence the children’s coping [85] although we did not find interventions in the early post-traumatic period involving siblings.

It has become clear to clinicians and researchers alike that alongside the specific nature of the traumatic events themselves, it is the subjective experience at the time of the traumatic event that determines the range of immediate posttraumatic reactions, as well as the degrees of recovery.

Each child’s exposure and reactions to a PTE are unique and there is no definite answer or explanation when understanding children’s adaptive capacity and resilience [86]. Moreover, qualitative studies exploring children’s own subjective experiences from their own perceptions are scarce [45], even though previous research suggested that youth can be effective and informed partners in the research process [87,88]. The wide range of children’s reactions to PTEs as well as the lack of understanding of children’s own perceptions and experiences in the aftermath of the trauma exposure points to the importance of developing a more thorough assessment of their experiences of coping and adaptation and how they are affected by their social milieu and relationships. Children’s coping abilities depend on the internal (feelings of self-efficiency) and external (e.g., family and social support networks) resources available in their ecological contexts [85,89] and their interaction with their proximal environment: family, school, and neighborhood [85]. Trauma research needs to shift the focus from children’s symptomology to exploring processes allowing children to respond in an adaptive manner within their environmental settings [90].

### 4.3. Limitations

Only two databases were used to search for articles, due to limited logistic resources. However, for scoping reviews the two databases that should be at least applied are MEDLINE and Embase. We searched PubMed using Mesh terms, which is equivalent to searching MEDLINE and searched Embase while excluding results of MEDLINE to avoid any duplication. A larger review including databases, such as APA Psyinfo is warranted to expand the results, and the small number of included studies emphasize the need for further rigorous studies in the field.

## 5. Conclusions and Future Directions

Early psychological interventions combining psychoeducation content for both children and parents on trauma reactions, as well as coping strategies to deal with peritraumatic distress, seem to be efficient in specific settings in preventing the development of enduring posttraumatic stress. Exploring children’s perspectives in this process is crucial, to have a better understanding of their adaptation processes and how they are affected by their social context and family relationships. Children’s abilities to adaptively process a traumatic event are influenced by their developmental stage and the environment in which their development takes place. When we are better able to consider and appreciate the combination of these factors, we are better positioned to offer an effective clinical intervention that can help reduce traumatic stress reactions, prevent PTSD and associated conditions, and decrease suffering and interference with subsequent development.

## Figures and Tables

**Table 1:** An overview of studies’ research characteristics.

First Author, Year	Study Design	Type of Intervention	Intervention Components	Time of Intervention after Traumatic Event	Traumatic Event	Target Population Characteristics	Age of Target Population
Berkowitz, 2011 [10]	RCT	CFTSI	Four sessions targeting either the caregiver/child alone or the caregiver and child together. The sessions involved different components, such as psychoeducation, questionnaires administration, teaching relaxation techniques and coping strategies.	Within 30 days.	Motor vehicle accident, sexual abuse, witnessing violence, physical assaults, injuries, animal bite, and threats of violence.	Telephone screening based on a report of one new distressing posttraumatic stress symptom after a PTE	7 to 17 years old
Hildenbrand, 2020 [49]	Prospective, Longitudinal Study	Pharmacological	Administration of Opioid medications	Within 2 weeks	Unintentional injury	Children who had an injury requiring inpatient treatment	8 to 13 years old
Nugent, 2010 [50]	RCT (double blind)	Pharmacotherapy	Intervention: group Propranolol Control group: placebo	12 h postadmission	Physical injury	Pediatric injury patients	10 to 18 years old
Rosenberg, 2018 [51]	RCT (double blind)	Pharmacotherapy	Intervention group: Acute Propranolol Treatment Control group: non-propranolol treatment	5 ± 8 days after the burn trauma	Burn injury	Children hospitalized for large burns	6–13 years at the time of the burn incident
Saxe, 2001 [52]	Experimental	Pharmacotherapy	Morphine	After Hospital admission for an acute burn	Burn injury	Children Hospitalized for acute burns	6–16 years old
Sharp, 2010 [53]	Retrospective review of medical records	Pharmacotherapy	Propranolol	Average of 2 days postburn	Burn injury	Children with severe burn injuries	Mean age = 7 years old
Stoddard, 2011 [54]	RCT (double blind)	Pharmacotherapy	Intervention group: Sertraline Control group: Placebo	After admission to the burn center for an acute burn or for reconstructive burn surgery (no mentioning of exact time)	Burn injury	English- and Spanish-speaking children admitted to a pediatric burn center for acute burns or reconstructive burn surgery	6 to 20 years old
Stoddard, 2009 [55]	Experimental	Pharmacotherapy	Morphine	Within days of admission	Burn injury	Children with acute burns hospitalized in a major pediatric burn center as well as their parents	12–48 -month-old
Hahn, 2019 [56]	Meta-analysis	CFTSI	Improving the capacity to self-observe and identify post traumatic reactions, recognize trauma reminders, learn strategies to manage trauma-related symptoms in both children and parents.	Following formal disclosure of abuse (no mentioning of exact time)	Sexual abuse Physical abuse Other PTEs	Child-Caregiver dyads	Majority 7 to 12 years
Uggla, 2016 [57]	RCT	Music therapy	The child in invited to listen, sing, and play different musical instruments, during which the child may choose to express different sensations, emotions, and thoughts	After children undergo the hematopoietic stem cell transplants (HSCT) (no mentioning of exact time)	HSCT	Pediatric recipients of (HSCT)	Up to the age of 16
Pityaratstian, 2009 [58]	Experimental	Cognitive-behavioral.	Children are taught different methods to deal with the psychological impact of trauma including hyperarousal and avoidance	57th day after the tsunami	Tsunami	Children in a tsunami-hit area	9 to 15 years old
Jordans, 2010 [59]	Cluster-Randomized Trial	Classroom based Psychosocial intervention	Cognitive behavioral therapy, experiential and creative-expressive therapy, and cooperative play	Ongoing war at the time of intervention	War	School-going children, in southwestern Nepal	11 to 14 years old
O’Callaghan, 2014 [60]	RCT	Family focused, psychosocial intervention	A youth life skill leadership program, narrative and fictional mobile cinema clips, and relaxation techniques covering a wide variety of techniques derived from cognitive behavioral therapy and psychoeducation; communication and conflict resolution skills building.	Ongoing conflict at the time of intervention	Violent conflict	War-exposed youth at risk of attack	7 to 18 years old
Tol, 2012 [61]	Cluster Randomized Trial	Classroom based Psychosocial intervention	Cognitive behavioral techniques and creative expressive components	Ongoing war at the time of intervention	War	School-based children affected by war	9 to 12 years old
Tol, 2008 [62]	A Cluster Randomized Trial	Psychosocial	Cooperative play, activities that help in trauma processing, and creative-expressive components.	Ongoing conflict at the time of intervention	Political violence	Children attending schools in violence-affected communities	6 to 11 years old
Wood, 2020 [63]	Case Reports	Hypnosis	Different hypnotic techniques to help the child review the traumatic event	2–3 days after the accident	Motor vehicle accident	Four pediatric patients presenting early distressing symptoms after an accident	2 to 15 years old
Zehnder, 2010 [64]	RCT	Psychological intervention	Four step process including reconstruction of the traumatic event, the creation of a trauma narrative using aids, identification of trauma-related appraisals, and psychoeducation.	Within 10 days after the child’s road accident	Road traffic accident	Children or adolescents receiving inpatient or outpatient medical treatment after a road traffic accident, who were fluent in German	7 to 16 years old
Phipps, 2012 [65]	RCT 171 patients and parents were assigned randomly to receive a child focused intervention; a child-parent intervention; or standard care	Complementary Intervention	Relaxation/imagery, humor therapy, and massage	Patients were recruited before admission for Stem Cell Transplantation (SCT)	SCT	Children under stem cell transplantation	6–18 years old
Stallard, 2006 [66]	RCT	Structured debriefing process	A structured debriefing process involving a detailed reconstruction of the accident, helping the child identify thoughts about the trauma and discuss related emotions after which information about trauma feelings was provided to normalize reactions and help in coping with common problems.	4 weeks	Road traffic accidents	Youth who attended the accident and emergency department	7 to 18 years old
Kenardy, 2008 [67]	RCT	Information-provision	Three booklets aimed at normalizing traumatic stress response	Within 72 h	Motor Accidents, sporting injuries falls,	Children and their parents after an accidental injury	7 to 15 years old
Kenardy, 2015 [68]	RCT	Web-Based early intervention	Booklet aimed at relieving and normalizing trauma reactions by providing resiliency strategies, coping skills, and psychoeducation.	Within 72 h of the accident	Accidental injury.	Youth with elevated initial distress post injury	7 to 16 years old
Cox, 2010 [69]	RCT	Web-based psychoeducation intervention	Booklet with information aiming at relieving, and normalizing trauma reactions, with incorporated cognitive-behavioral practical tools, and resiliency strategies for both parents and children.	Within 2 weeks	Unintentional Injury	Children recruited from pediatric surgical units	7 to 16 years old
Kassam- Adams, 2011 [70]	RCT	Stepped Preventive Care	Two sessions incorporating assessment and psychoeducation with both parents and children	Baseline assessments completed within 2 weeks post- injury	Unintentional injury	Hospitalized injured children	8 to 17 years old
Marsac, 2013 [71]	RCT	Web-based psycho-educational intervention	Information and psychoeducation on trauma including videos, interactive features, and care plans involving both parents and children and aimed at increasing parental perceived self-efficiency in supporting their children.	Injury within the past 60 days	Pediatric injury	Children with injuries requiring medical attention and their parents	6 to 17 years old
Haag, 2020 [72]	Multi-site RCT	CARE intervention: Coping with Accident Reactions	Psychoeducation, trauma narrative, and coping strategies involving both parents and children.	6–8 days postaccident	unintentional injury (burns, animal bite, road accident)	Children requiring inpatient or outpatient treatment	1 to 6 years old
Kassam- Adams, 2016 [73]	RCT	Coping coaching intervention	An interactive, game-like format, aimed at teaching children adaptive coping strategies	Within 2 weeks	Acute medical event	Children admitted to the hospital for an acute medical event	8 to 12 years old
Melnyk, 2004 [74]	A Cluster Randomized Trial	The COPE Intervention program	Psychoeducation and support for parents during and after admission	6–16 h after Pediatric intensive care unit admission	Respiratory, neurological, hematologic or cardiac problems, accidental trauma, infections, ingestions), or other causes	2- to 7-year-old children and their mothers urgently hospitalized in the pediatric intensive care unit of 2 hospitals.	2 to 7 years old

**Table 2:** An overview of the studies’ findings and statistical significance.

First Author, Year	Targeted Outcomes	Findings	Statistical Significance
Berkowitz, 2011 [10]	PTSD in youth at 3 months follow-up	Significantly lower posttraumatic and anxiety scores in the intervention group and decreased prevalence of PTSD at follow-up in the intervention group.	Significant group differences between CFTSI and comparison group at follow-up in relation to PTS (*p* = 0.04) and anxiety (*p* = 0.009) scores CFTSI significantly reduced the odds of partial or full PTSD by 73% (*p* = 0.008)
Hildenbrand, 2020 [49]	PTSS in children at 12 weeks follow-up	Morphine did not mediate the relationship between pain and post-traumatic stress	*p* = 0.85, 95% confidence interval, −0.09–0.07
Nugent, 2010 [50]	PTSD in children at 6 weeks follow up	Girls who had propranolol reported more PTSD symptoms compared to girls receiving placebo, however, a non-significant trend was reported among boys	$\mathrm{More}\;\mathrm{PTSD}\;\mathrm{symptoms}\;\mathrm{in}\;\mathrm{girls}\;\mathrm{receiving}\;\mathrm{propranolol}\;\mathrm{compared}\;\mathrm{to}\;\mathrm{placebo}\;R^{2}$$\;=\;0.44,\;\mathrm{and}\;\mathrm{insignificant}\;\mathrm{trend}\;\mathrm{among}\;\mathrm{boys}\;R^{2}$ = 0.32
Rosenberg, 2018 [51]	PTSD, anxiety, and depression 7 years post burn on average	No significant difference in the prevalence of PTSD, anxiety and depression between both groups.	PTSD: (Chi-square = 0.00, *p* = 0.97). Anxiety: (Chi-square = 0.31, *p* = 0.58) Depression: (Chi-square = 0.00, *p* = 0.99)
Saxe, 2001 [52]	PTSD in children at 6 months follow up	Significant association is found between the dose of morphine received during hospitalization and the reduction in PTSD at 6-month follow-up.	r = 0.44, *p* < 0.05
Sharp, 2010 [53]	Acute Stress Disorder (ASD) among children	Propranolol did not influence the risk for ASD	$X^{2}$ = 0.456, *p* = 0.4996, Fisher’s exact test, *p* = 0.3702
Stoddard, 2011 [54]	PTSD in children at 8, 12 and 24 weeks follow-up	According to the parent’s report, sertraline was moderately more effective in preventing PTSD symptoms than placebo	Decrease in parent-reported symptoms over 8 weeks (−4.1 vs. −0.5, *p* = 0.005), over 12 weeks (−4.4 vs. −1.2, *p* = 0.008), and over 24 weeks (−4.0 vs. −0.2, *p* = 0.017).
Stoddard, 2009 [55]	PTSD in children at 1 month, 3 and 6 months	Morphine may be correlated with a decreasing number of post-traumatic stress symptoms	Correlation between Morphine dose and amount of decrease in PTSD symptoms (r = −0.32) The correlation between morphine dose and amount of decrease in arousal cluster of the CSDC (R = −0.63, *p* < 0.05)
Hahn, 2019 [56]	PTSD in children and caregivers at 3 months follow-up	The intervention was associated with significant changes in children’s and caregiver’s PTSS	Hedge’s g = 1.17, Child-rated; g = 0.66, caregiver-rated
Uggla, 2016 [57]	Physiological parameters (heart rate, saturation, blood pressure) among children 4 to 8 h after the intervention	Lower heart rate in patients suggesting Decreased levels of stress and potentially decreased risk of PTSD development according to authors	Music therapy group had reduced heart rates compared to control group *p* < 0.001
Pityaratstian, 2009 [58]	Post-traumatic symptoms at 2 weeks follow-up using the Children’s Impact of Events Scale (CRIES-13).	Significant reduction in CRIES scores when the children were already prone to develop PTSD, but significant increase in the scores in other children.	Significant decreases in the CRIES + ve group (*p* = 0.00 in all), while significant increase in the the CRIES-ve group (*p* = 0.00 in all).
Jordans, 2010 [59]	Psychiatric symptoms among children post-intervention (depression, anxiety, posttraumatic stress disorder), psychological difficulties, resilience indicators (hope, prosocial behavior), and function impairment	The intervention did not reduce posttraumatic symptoms, however, it led to positive results for other indicators	For Child PTSD Symptom Scale: T (df); *p* = −0.06 (323) 0.951
O’Callaghan, 2014 [60]	PTSD, depression and anxiety symptoms, conduct problems, prosocial behavior among children at 3 months follow-up	Participants reported significantly fewer PTSS compared to controls	Cohen’s d = 0.40
Tol, 2012 [61]	PTSD, depressive, and anxiety symptoms in children at 1 week and 3 months follow-up	No significant effects on primary outcomes	**Boys: (Treatment vs. waitlist)** PTSD: *p* = 0.997 Depression: *p* = 0.974 Anxiety: *p* = 0.38 **Girls: (Treatment vs. waitlist)** PTSD: *p* = 0.023 Depression: *p* = 0.343 Anxiety: *p* = 0.506
Tol, 2008 [62]	PTSD symptoms, trauma idiom, anxiety symptoms, depressive symptoms, functional impairment, hope, aggression, coping, social support, and family connectedness at 1 week and 6 months follow-up	The intervention decreased posttraumatic stress symptoms and instilled hope. However, it did not reduce traumatic-stress related symptoms, depressive symptoms, anxiety symptoms, or functional impairment	Posttraumatic stress disorder symptoms (mean change difference,2.78; 95% confidence interval [Cl], 1.02 to 4.53) and hope (mean change difference, −2.21; 95% Cl, −3.52 to −0.91)
Wood, 2020 [63]	PTSD in children	Hypnosis led to improvements in all patients after one or more Sessions	NA
Zehnder, 2010 [64]	PTSD symptoms, Depression, Behavior in children at 2 or 6 months follow-up	Children in both the intervention and control groups had no significant differences in posttraumatic symptoms	No significant between group differences were found at any time point for PTSS (T1: t = 0.81, *p* = 0.42; T2: t = 0.58, *p* = 0.57), depressive symptoms (T1: t = −0.34, *p* = 0.74; T2: t = −0.36, *p* = 0.72) or behavioral problems (T1: t = −0.01, *p* = 0.99; T2: t = −0.40, *p* = 0.69).
Phipps, 2012 [65]	Depression and posttraumatic stress, HRQL, among children at 24 weeks follow-up	Significant improvements in all outcomes for all three groups and no statistical differences between intervention arms for any of the measured outcomes.	PTSS declined significantly from admission to week + 24 (F = 21.3, *p*, 0.001). No difference between groups (F = 0.9, *p*. 0.3), and no intervention effect (F = 0.8, *p*. 0.4)
Stallard, 2006 [66]	Self-reported psychological distress and diagnostic criteria for PTSD at 8 months follow up	No additional significant gains of the intervention, as both groups reported improvements during follow-up	No significant difference between The experimental or control $\mathrm{Groups}\;(X^{2}\;=\;0$.263, df = 1, *p* = 0.608).
Kenardy, 2008 [67]	Anxiety and PTSS symptoms in both children and parents at 1 and 6 month follow-up	Anxiety reduced in children at one-month follow-up, posttraumatic intrusion symptoms among parents, and overall PTSS, at 6 month follow-up	Treatment condition had an effect on children’s total anxiety as measured by the SCAS (F (adj df = 1.82), 142.21) = 2.14, *p* = 0.01). Treatment condition had an significant effect on parent’s IES intrusion symptoms (F (adj df) = 1.64, 99.72) = 2.09, *p* = 0.02).
Kenardy, 2015 [68]	PTS Reactions in children at 6 month follow-up	Children in the control group had significantly increased trauma symptoms at 6-month follow-up, only when initial distress was high	d = 0.94, *p* = 0.008
Cox, 2010 [69]	PTSD symptoms, anxiety, depression in children and parents at baseline, 4–6 weeks, and 6 months follow-up	The intervention group reported improved symptoms in youth who had higher baseline trauma scores	Treatment condition had an effect on child anxiety F (1, 52) = 4.18, *p* < 0.05, d = –0.34 (CI = –0.86 to 0.19).
Kassam- Adams, 2011 [70]	PTSD in children at 6 months follow-up	No reduction in PTSD, depression severity, or increase in quality of life in the intervention group in comparison to the control group	No significant group by time $\mathrm{Interaction}\;(\mathrm{Wald}\;X^{2}$ = 4.15; df = 2; *p* = 0.13)
Marsac, 2013 [71]	Posttraumatic stress in both children and parents at 6 weeks follow-up	The Intervention had no significant impact on parent knowledge or PTSS	Child report and PTSS at 6 months follow-up (SD): 6.05 (7.45). T value (−0.6), F value (0.02)
Haag, 2020 [72]	PTSD symptoms and severity; functional impairment, and behavioral problems in children at 3 and 6 months postinjury	The intervention had a significant effect on PTSS severity at follow up	Reduction of PTSS between intervention and control at 3 months follow-up (M = 17.30, SD = 13.94, range 0–52; mean difference −6.97, 95% CI: −14.02 to 0.08, adj. 0.055, d = 0.51).
Kassam- Adams, 2016 [73]	Persistent posttraumatic stress in children at 6 and 12 weeks follow-up	The intervention could prevent persistent posttraumatic stress	Change in PTSS severity from baseline to 6 weeks (d = −0.68) or 12 weeks (d = −0.55)
Melnyk, 2004 [74]	Maternal anxiety, low mood, maternal beliefs, parental stress, and parent involvement in their children’s care, child adjustment, at 1, 3, 6, and 12 months follow up	Mothers receiving COPE program had increased maternal functioning and coping resulting in better adjustment in children.	$\mathrm{One}-\mathrm{year}\;\mathrm{post}\;\mathrm{discharge}\;\mathrm{there}\;\mathrm{was}\;a\;\mathrm{higher}\;\mathrm{percentage}\;\mathrm{of}\;\mathrm{control}\;\mathrm{group}\;\mathrm{children}\;(25.9\%)\;\mathrm{with}\;\mathrm{significant}\;\mathrm{behavioral}\;\mathrm{symptoms},\;\mathrm{compared}\;\mathrm{with}\;\mathrm{COPE}\;\mathrm{group}\;(2.3\%)\;(X^{2}=\;$1, Df = 9.36, *p* < 0.01).
